# The challenge of university teaching in times of the COVID-19 pandemic

**DOI:** 10.1590/0034-7167.2023760201

**Published:** 2023-02-06

**Authors:** Francisco Javier Castro Molina

**Affiliations:** I Universidad de La Laguna, Escuela de Enfermería de Nuestra Señora de Candelaria. Canary Islands, Spain.

This planet’s history has been a long and sometimes tortuous one. To date, a total of five disappearances of life on Earth have been distinguished: the great oxidation; the Ordovician-Silurian extinction; the Devonian extinction; the Permian-Triassic extinction; the Triassic-Jurassic extinction; and the Cretaceous-Tertiary extinction. The causes? Very varied and diverse: anoxia phenomena in the marine environment, competition between species, climate change, volcanology, meteorite collisions, changes in the orbit or magnetic field, or attacks by microorganisms that have managed to consolidate epidemics^(1)^.

Nobody would have imagined that in 2020 what happened would happen. The world’s nurses were preparing to celebrate the ‘Year of Florence Nightingale’, their year, and everything was interrupted by a pandemic, similar to the ‘1918 Flu’, which stopped the entire planet. “Death” did not stop its work, which was facilitated by the end of a war never seen until today and health conditions considerably different from those we have today^(2)^. Despite having much more resources, health professionals working in the network of the Spanish national health system found themselves defending the population with all their might, and professors, who until then worked in university classrooms, had to go to a ‘distance’ environment to continue monitoring and teaching their students.

We cannot forget that the yesterday and today of nurses have always been marked by constant changes that forced a repeated adaptation to new moments^(3)^. For our generation, the COVID-19 pandemic was an entirely new situation that required us to adapt, generating changes in many of the daily environmental stimuli: changes in routines, changes in relationships with family and friends and, of course, changes in the learning environment. On the other hand, our priorities have changed, and have changed, where people have lost their jobs, or seen them in danger, with the result that their basic needs for security and stability are not covered. But, above all, people felt a permanent threat, in which the alert situation was a constant uncertainty about the future, making living together a complicated path, the result of a confinement that was difficult for us to go through and understand. This feeling carried a significant emotional cost for students, which materialized in sleep disorders, eating disorders or simply behavioral disorders, situations that encouraged the emergence of considerable active negativism in the face of the tasks they had to face and in the face of the gain of a strange social dynamic. All this made our situation difficult as professors, becoming ‘full-time professors’ with a student body that was afraid, with a student body that lived in uncertainty, with a student body that needed to be monitored. This follow-up, both of students and their families, had to be done from educational centers, where we had to facilitate the teaching-learning process not only in the academic-curricular aspect, but also affectively, transforming ourselves into an ‘academic super-father’ who sought to full-time support and monitor the educational process. This occurred in a scenario where not all students had the same context, means, space or adaptability.

For us, university professors, coordination was essential to balance the number of tasks assigned to students, define the number of activities that each student in our charge had to face and the maximum time of daily dedication, and also assess the degree of difficulty and the different paces in the learning process^(4)^. We came to agree on a series of priority guidelines that we must follow as professors: promoting students’ autonomy in carrying out academic tasks; offer varied activities, not only in the instrumental areas, seeking a comprehensive education that includes creative activities, and taking advantage of communication through ICT; encourage the expression of emotions and feelings in this situation, offering different tasks in which they can express themselves (draw how they feel, write a diary, letters or post about their experiences in those days), to send to their family members, professors or classmates; diversify tasks according to each student’ needs and, of course, their families’: carry out basic tasks with the minimum criteria to be carried out by all, complementary tasks and voluntary extension tasks; and, finally, encourage students to intersperse recreational activities with curricularly necessary teaching activities^(5)^. There were many tips, which in this monitoring, professors sent their students such as: avoiding continuous overexposure to the media and news about the virus, the state of alert and all those ‘toxic messages’ that bother us every day; maintaining a schedule and a daily routine, seeking time stability from Monday to Friday; encouraging students to maintain social contact with their peers, by telephone, video call, through social networks which, at that time, became our windows to the world; maintaining the tranquility that we transmit with our good work, although many times we do not even have it as people. Moreover, professors should be, at all times, an element of help and monitoring beyond the curriculum, helping students to live this stressful moment with serenity, to know how to live together in a different way, in a way unknown until now and to recognize and thank each of the social expressions of solidarity that gradually emerged in order to be able to face, in some way, this tragic event^(6)^.

But even so, life went on, and little by little we recovered a ‘strange normality’, we recovered a day to day that was not like that day to day that was in March 2020, because from that moment on we became completely different men and women, with different fears, with a different way of acting, with a different perspective from the one that followed us in past times. Toba Beta said, “a positive person flows with the unexpected”, and this has happened to us, we flowed and managed to overcome this ‘obstacle’ that wanted to blur our world and that helped us design a different one.
